# Profiling of naïve and primed human pluripotent stem cells reveals state-associated miRNAs

**DOI:** 10.1038/s41598-020-67376-w

**Published:** 2020-06-29

**Authors:** Benjamin T. Dodsworth, Klas Hatje, Maria Rostovskaya, Rowan Flynn, Claas A. Meyer, Sally A. Cowley

**Affiliations:** 1 Sir William Dunn School of Pathology, University of Oxford, South Parks Road, Oxford, OX1 3RE UK; 2 Roche Pharma Research and Early Development, Roche Innovation Center Basel, Grenzacherstrasse 124, 4070 Basel, Switzerland; 3 Epigenetics Programme, Babraham Institute, Cambridge, CB22 3AT UK; 4 Censo Biotechnologies, Roslin Innovation Centre Charnock Bradley Building, Easter Bush Campus, Roslin, EH25 9RG UK

**Keywords:** Pluripotency, miRNAs, Pluripotent stem cells

## Abstract

Naïve human pluripotent stem cells (hPSC) resemble the embryonic epiblast at an earlier time-point in development than conventional, ‘primed’ hPSC. We present a comprehensive miRNA profiling of naïve-to-primed transition in hPSC, a process recapitulating aspects of early in vivo embryogenesis. We identify miR-143-3p and miR-22-3p as markers of the naïve state and miR-363-5p, several members of the miR-17 family, miR-302 family as primed markers. We uncover that miR-371-373 are highly expressed in naïve hPSC. MiR-371-373 are the human homologs of the mouse miR-290 family, which are the most highly expressed miRNAs in naïve mouse PSC. This aligns with the consensus that naïve hPSC resemble mouse naive PSC, showing that the absence of miR-371-373 in conventional hPSC is due to cell state rather than a species difference.

## Introduction

Naïve human pluripotent stem cells (hPSC) are cells in vitro resembling the inner cell mass of human embryonic day (E) 6–7 preimplantation blastocysts^1,2^. Conventional hPSC are not considered naïve but instead are referred to as primed, since they resemble a later stage found in the post-implantation epiblast^2–8^. A plethora of methods currently exist to generate naïve hPSC^9^, which can be evaluated using various molecular markers, particularly gene and transposon expression profiles, cell surface protein expression and genome-wide DNA demethylation (reviewed in^10^).

MicroRNAs (miRNAs) are a class of small non-coding RNAs (∼ 22 nucleotides) which inhibit complementary mRNAs by binding to the 3′ untranslated region (UTR) and either flagging it for degradation or blocking translation. Early miRNAseq experiments in mouse embryonic stem cells (mESC) have shown that pluripotent stem cells have a miRNA expression patterns dominated by a few miRNAs which have been defined as the ESC-specific cell cycle-regulating (ESCC) miRNAs. ESCCs have the seed sequence AAGUGC and make up 20–50% of all miRNAs in mESC^11–15^. ESCC miRNAs are specifically expressed in naïve and primed pluripotent states and are downregulated upon differentiation. Upregulation of these miRNAs in somatic cells has also been associated with proliferation and cancer in mouse and human (reviewed in^16^).

In murine cells both in vivo and in vitro, there is a switch in dominant ESCC miRNA expression in pluripotent cells from the preimplantation/naïve miR-290 family to the postimplantation/primed miR-302 family both containing the seed sequence AAGUGC^17–21^. However, the miR-290 family does not exist in the same genomic context in the human. The human homolog, the miR-371-373 cluster, is reported to be variably, if at all, expressed in conventional human PSC and this discrepancy has previously been viewed as a key difference between the two species^13,14,22^. Conversely, high expression of the miR-302 family has been reported as a key marker of primed human ES and iPS cells^13,14,23^ and mouse EpiSC^11^.

Here we present a comprehensive miRNAseq dataset of naïve, intermediate and primed hPSC. This dataset reveals that the miRNA expression profiles of naïve and primed pluripotent states differ considerably and thus we add to the growing repertoire of molecular markers of these distinct developmental stages. We identify miRNA markers of the human naïve (miR-143-3p, -22-3p) and primed states (miR-363-5p, several members of the miR-17 family, miR-302 family) and show that the miR-371-373 cluster is highly expressed in naïve hPSC.

## Materials and methods

Reagents were from ThermoFisher unless stated otherwise. More detailed descriptions are in the Supplementary Experimental Procedures.

### Cell culture

The origins of the iPSC lines used in this study are described in Table S2 (all derived previously with informed consent from National Health Service, Health Research Authority, NRES Committee South Central, Berkshire, UK, REC 10/H0505/71). The naïve hESC lines HNES1 and HNES2 cells were previously derived^24^ with informed consent (North East-York Research Ethics Committee Approval number 04/MRE03/44) under licence from Human Embryology and Fertilisation Authority. See hPSCReg for further details. Naïve cells were generated and grown on irradiated (30 gy) CF1 mouse embryonic fibroblasts (MEFs) (Millipore; PMEF-CFL) as previously described^25^ with minor adaptations from subsequent publications: IM-12 from the original Theunissen et al*.* was omitted in 4iLA^10^; 70% media changes were implemented^26^. t2iLGoY for HNES cells was prepared as previously described^24^. Naïve cells were split 1:1–1:3 every 3–4 days cells by PBS washing, adding 1 mL of Accumax (A7089; Sigma), incubating at 37 °C for 4 min and dissociating by pipetting with a P1000. To prime naïve cells, 1–5 × 10^4^ cells/cm^2^ were plated onto a well of a six well plate (precoated with geltrex for > 1 h). Medium was switched to E8 after 48 h. Transition and analysis was performed as previously described^8,27^.

### RT-qPCR and miRNAseq

RNA extractions from cell pellets (equal or less than 1 × 10^6^ cells) were performed using the RNeasy mini kit (74,104; Qiagen) or the miRNeasy mini kit (for the analysis of miRNAs; 217,004; Qiagen), following the manufacturer’s protocol. RT-qPCR was performed using the High Capacity RNA to cDNA kit (4,387,406), TaqMan™ primer-probes (4,331,182) and TaqMan™ Gene Expression Master Mix (4,369,016) according to the manufacturer’s protocol. For miRNA RT-qPCR, the TaqMan advanced miRNA assay cDNA synthesis kit (A28007) was used in conjunction with TaqMan Advanced miRNA Assays (A25576) and TaqMan Fast Advanced Master Mix (4,444,557) according to the manufacturer’s instructions. For miRNAseq, purified total RNA was sent to EA | Q^2^ Solutions (5,927 S. Miami Blvd., Suite 100, Morrisville, NC 27,560, US) who performed library preparation and miRNA sequencing.

Statistical analysis was performed using GraphPad Prism 7. Error bars indicate the standard deviation, and statistical tests performed are detailed in the figures. Statistical significance was defined as ns = *P* > 0.05; * = *P* < 0.05; ** = *P* < 0.01; *** = *P* < 0.001; **** = *P* < 0.0001.

## Results and discussion

### Transition of naïve hPSC to primed hPSC

**Figure 1:**
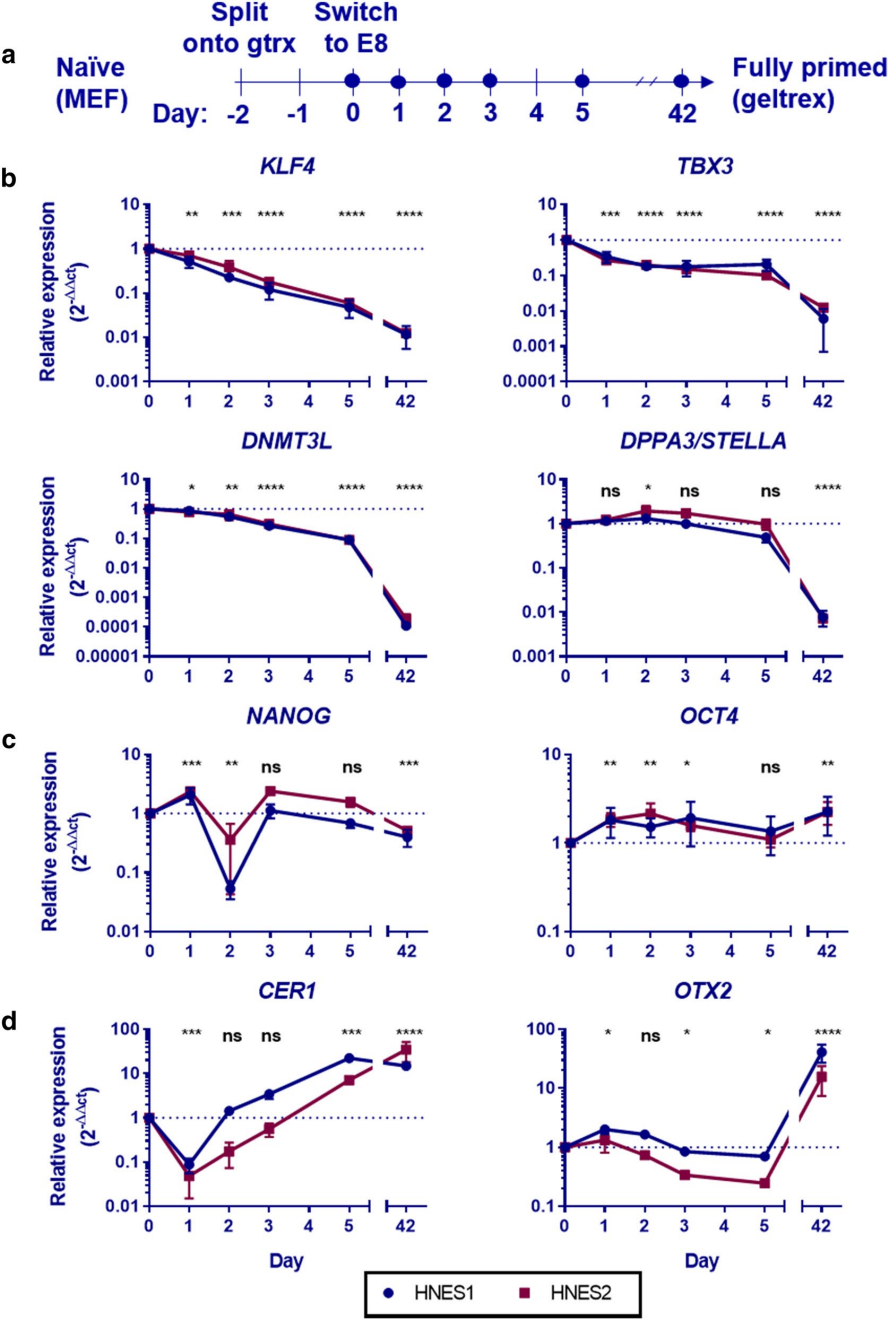
Gene expression during naive-primed transition. (**a**) Schematic of the experiment, dots represent when samples were taken. Expression of (**b**) marker genes of the naïve state, (**c**) shared pluripotency and (**d**) primed state are shown across the time course. Data normalised first to housekeeping genes (B2M, RPL13A), then to day 0 using the 2^−ΔΔCt^ method. Error bars indicate the standard deviation between 3 biological replicates for each cell line. A two-tailed ratio paired t-test of the 2^−ΔCt^ values of all 6 replicates informed significance. HNES1 is marked blue, HNES2 red.

To investigate miRNAs involved in the transition of naïve hPSC to primed hPSC, the effectiveness of the in vitro transition process itself was first assessed, using the directly derived naïve HNES1 and HNES2 cell lines. The transition was instigated in three separately cultured replicates of each of HNES1 and HNES2 naïve cells, plating them on geltrex and switching medium to E8 after 48 h^27^, thereby removing potential variability introduced if splitting the cells (schematic, Fig. 1a). The time points were chosen to capture the initial exit from naïve pluripotency, with a final late time point to ensure complete priming of the cells. Day 42 was chosen for the final time point since the cells had by then gained the ability to differentiate to the three major lineages using standard primed protocols^8^ (Supplementary Fig. S1).

When HNES cells were transitioned to the primed state, naïve marker genes were downregulated, though not all simultaneously (Fig. 1b). *KLF4* was significantly downregulated within 24 h and continued to decline. *TBX3* was significantly downregulated within 24 h, maintaining these levels until at least day 5, but was further downregulated when the cells reached the fully primed state. *DNMT3L* was slowly but significantly downregulated during the first 5 days and was heavily downregulated by the time the primed state was acquired. *DPPA3* levels did not change during the first 5 days but it was heavily and significantly downregulated when the cells were fully primed. Markers of shared pluripotency (*NANOG* and *OCT4*), active in both states, did not show a change in direction overall during the transition (Fig. 1c). The primed marker gene *CER1* was upregulated within the first 5 days, whereas *OTX2* was only heavily upregulated upon acquisition of the primed state (Fig. 1d). Together, these gene expression patterns in combination with the ability to differentiate to three lineages using standard primed protocols show that naïve HNES1 and HNES2 cells were successfully transitioned to primed cells.

Prior to this publication, the naïve to primed transition has been used extensively as a model of development in the mouse reviewed in^28^ and only recently has been used in the human^8^. To the best of our knowledge, the miRNAome of human naïve to primed transition has not been previously described.

### miRNAseq reveals state-associated miRNAs

**Figure 2:**
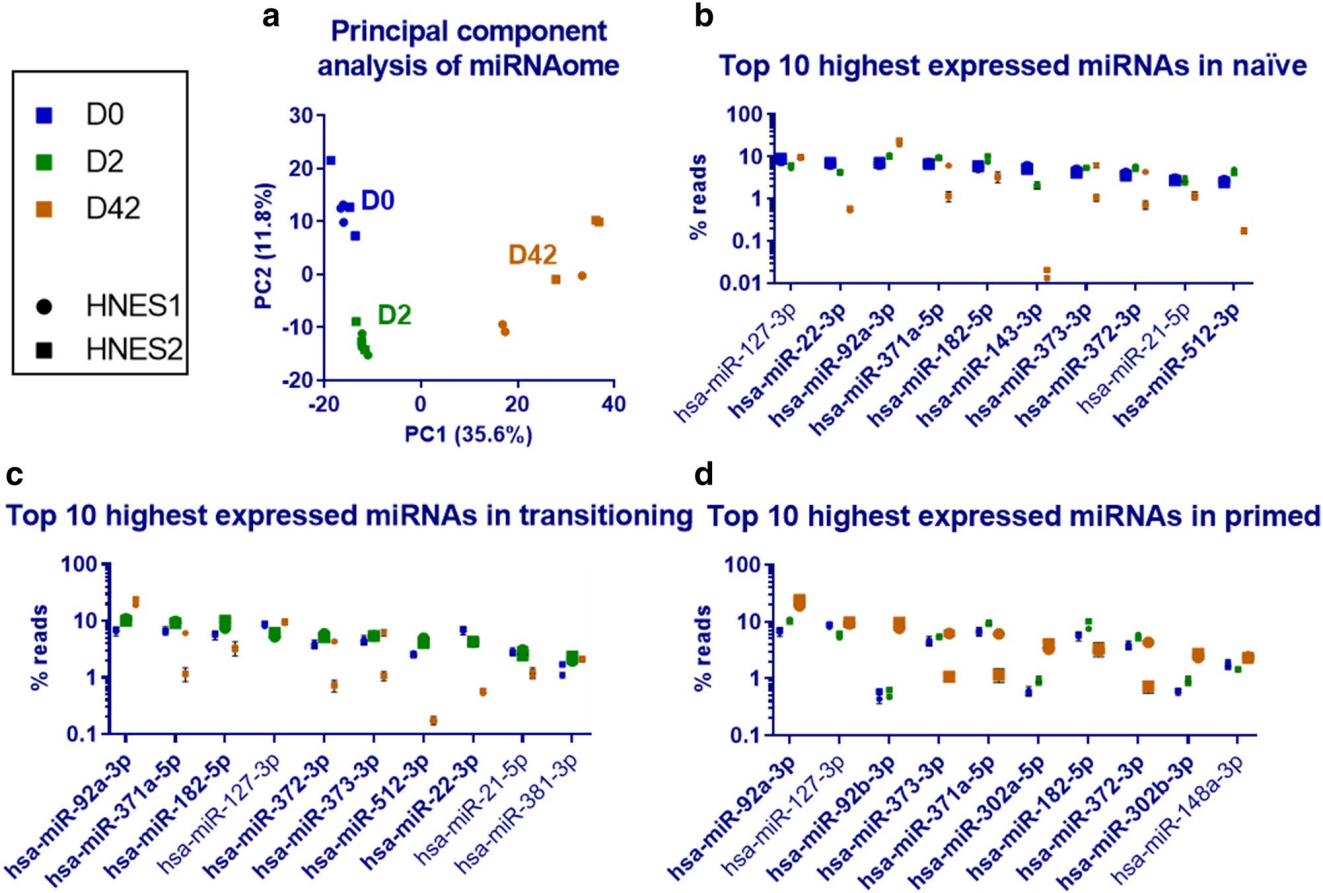
The miRNAome of naïve, transitioning and primed cells. miRNAseq was performed using samples from Fig. 1. (**a**) A principal component analysis was performed on the miRNAome alone. The symbol shape distinguishes HNES1 and HNES2 cells; samples from D0 are blue, D2 green and D42 orange. (**b**–**d**) The top 10 most highly expressed miRNAs in naïve, transitioning and primed cells are shown in separate graphs. The expression at the particular timepoint is shown by large symbols, other time points are included for comparison (smaller symbols). miRNAs written in bold are further investigated as potential markers in Fig. 4. Each symbol is the mean of 3 replicates ± standard deviation. No error bars indicate the symbol is larger than the error bars. HNES1 and HNES2 cells are shown as separate symbols (round or square, respectively).

Total RNA samples from naïve cells cultured above (HNES1 and HNES2, each cultured separately in biological triplicates) at day 0, during the transition (day 2) and when fully primed (day 42), were analysed by miRNAseq. Principal component analysis (PCA) shows a clear separation between naïve, transitioning, and primed cells, indicating that the miRNAome alone is sufficient to distinguish between states (Fig. 2a). Cells at day 0 and day 2 form separate tight clusters, whereas primed cells at day 42 are more diffuse. This is likely drift due to the extended culturing in separate wells for 42 days.

We detected 560 miRNAs in naïve, 573 in transitioning, 589 in primed cells (with a cut-off of at least 1 rpm). The top 10 highest expressing miRNA of each state collectively make up 54%, 59%, 61% of the miRNAome of naïve, transitioning and primed cells, respectively. miRNA-22-3p, -143-3p and -512-3p are amongst the most highly expressed in naïve but not primed cells. Conversely, miRNA-92b-3p, -302a-5p and -302b-3p were heavily expressed in primed but not naïve cells. (Fig. 2b–d).

The miR-371-373 cluster is amongst the highest expressing miRNAs in the naïve state and heavily downregulated in the primed state of HNES2 but not HNES1 cells (Fig. 2b–d). We found generally little variability between cell lines (59 differentially expressed miRNAs in naïve, 101 in primed with at least twofold average difference). Of the 101 miRNAs with at least twofold average difference in the primed state, 16 were significantly different (*P* < 0.01). This includes 6 members of the miR-371-373 cluster.

**Figure 3:**
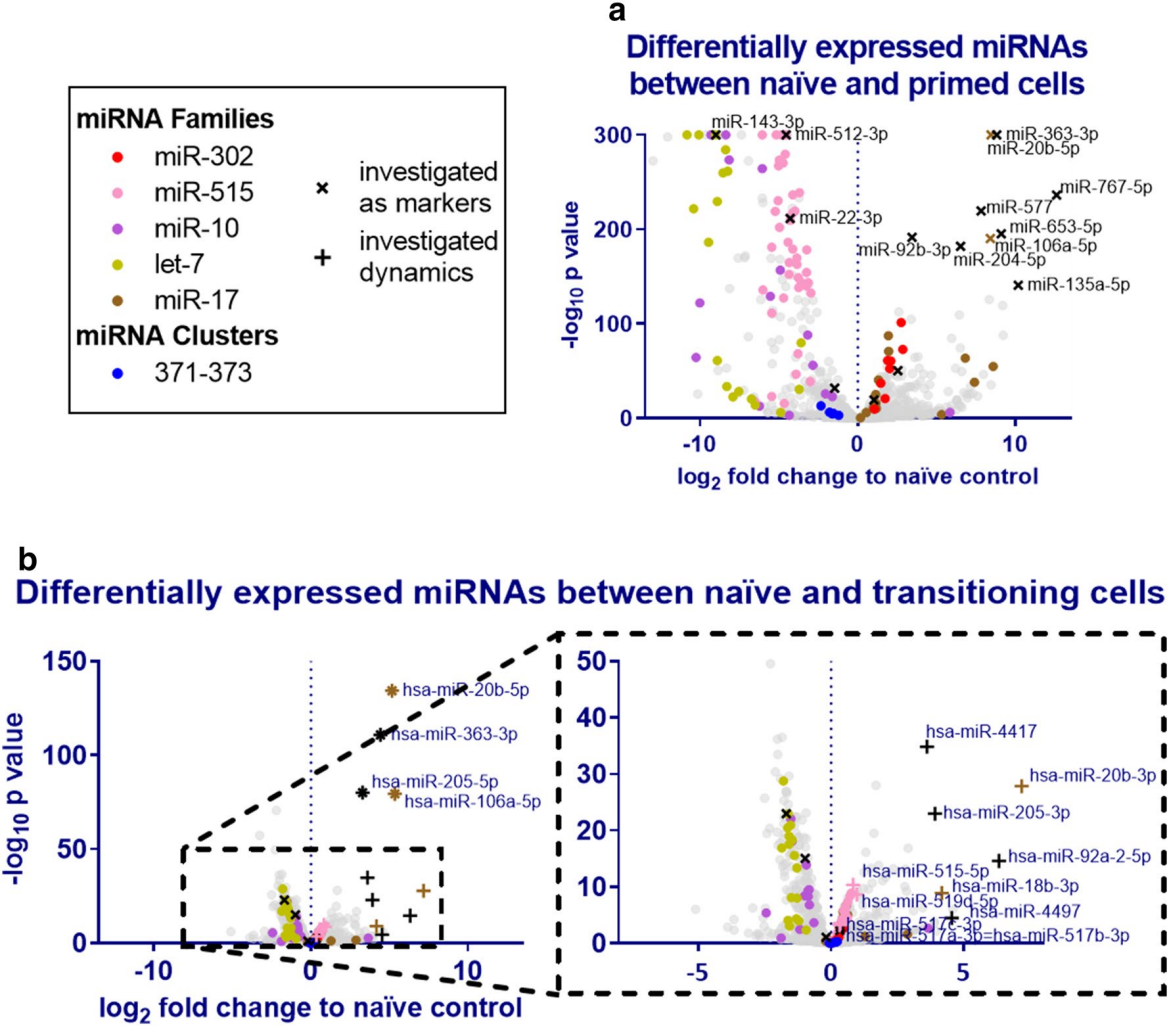
Changes in the miRNAome of naïve, transitioning and primed cells. miRNAseq was performed using samples from Fig. 1. Changes in the miRNAome from naïve to primed (**a**) and from naïve to transitioning (**b**) are shown in volcano plots. The − log10 *p* value is calculated for all 6 replicates. miRNAs of highly differentially expressed families or clusters are marked by colour. Highly and differentially expressed miRNAs are further investigated as potential markers in Fig. 4.

Figure 3a shows miRNAs differentially expressed between naïve and primed cells. Many miRNAs change between naïve and primed states (with at least twofold difference: 210 up-, 245 downregulated and 219 unchanged). Of note, members of the miR-302 and miR-17 family (in particular miRNA-20b-5p and -106a-5p) as well as miRNA-363-3p were highly upregulated in the primed state. In contrast, the miR-515, miR-10 and let-7 family miRNAs were downregulated in primed cells. The miR-371-373 cluster appears downregulated albeit with lower confidence due to variability between cell lines.

As in the mouse^21^, we have observed a switch of ESCC miRNA expression. The miR-371-373 cluster (the human homolog of the miR-290 family in the mouse) dominates expression in the naïve state, whereas the miR-302 family becomes dominant in primed cells. This explains a phenomenon which has puzzled scientists studying the evolution of miRNAs^22^.The miR-371-373 cluster is indeed highly expressed—just not necessarily in the primed hPSC previously assessed by small RNA sequencing.

Corroborating our results, a recent publication reported high expression of the miR-371-373 cluster in naïve Theunissen et al*.* 5iLAF cells using a single cell small-RNA sequencing approach, albeit only using one cell line^29^. We show that the expression of the miR-371-373 cluster becomes variable between lines in the primed state (with HNES1 showing high expression of 4–6% of the total miRNAome contrasting with HNES2 showing only 0.7–1%). This variability in expression of the miR-371-373 cluster has been previously reported for conventional primed ES cell lines in the literature^22^, with some publications implying no expression^13,30^. Our data confirms that the cluster is variable in primed cells but is universally highly expressed in naïve.

### MiRNAs in naïve to primed transition

Next, we focussed on the transition from the naïve to the primed state. At day 2, the miRNA profile remarkably already noticeably changed its miRNA expression profile (Fig. 2a). The exit from the naïve state is characterised by an immediate upregulation of miRNA-363-3p and miRNA-205-5p as well as miR-17 family members miR-18b-3p, -20b-5p, -20b-3p, -106a-5p.

Intriguingly, the miR-515 family is also heavily downregulated by day 42 (Fig. 3b). Both the miR-515 family and miR-371-373 cluster share the seed sequence AAGUGC and are located on the primate-specific chromosome 19 miRNA cluster. Expression of primate-specific chromosome 19 miRNA cluster miRNAs has been linked to pluripotency and cancer^31^. In contrast, the miR-302 family, that also contain the same seed sequence, are upregulated at day 42.

Due to the weak regulatory impact of single miRNA, a Gene Set Enrichment Analysis (GSEA) was performed to investigate if these miRNAs play a functional role. The GSEA was set up to find which messenger RNAs have an enrichment of in silico binding sites of day 2 upregulated miRNAs (Table S1). Due to the large amount of in silico binding partners of miRNAs and high frequency of false positives, the analysis is statistically not very powerful and the threshold for discovery is commonly set at a false discovery rate of 0.25 and below^32^. Once adjusted for gene set size and multiple hypothesis testing, the only significant target of these miRNAs is TBX3, a naïve pluripotency network transcription factor which is downregulated during the naïve to primed transition (Fig. 1b), cautiously suggesting that the upregulated miRNAs may play a role in the transition process. Further work is required to investigate the targets of these miRNAs.

### Validation of state-associated miRNAs

**Figure 4:**
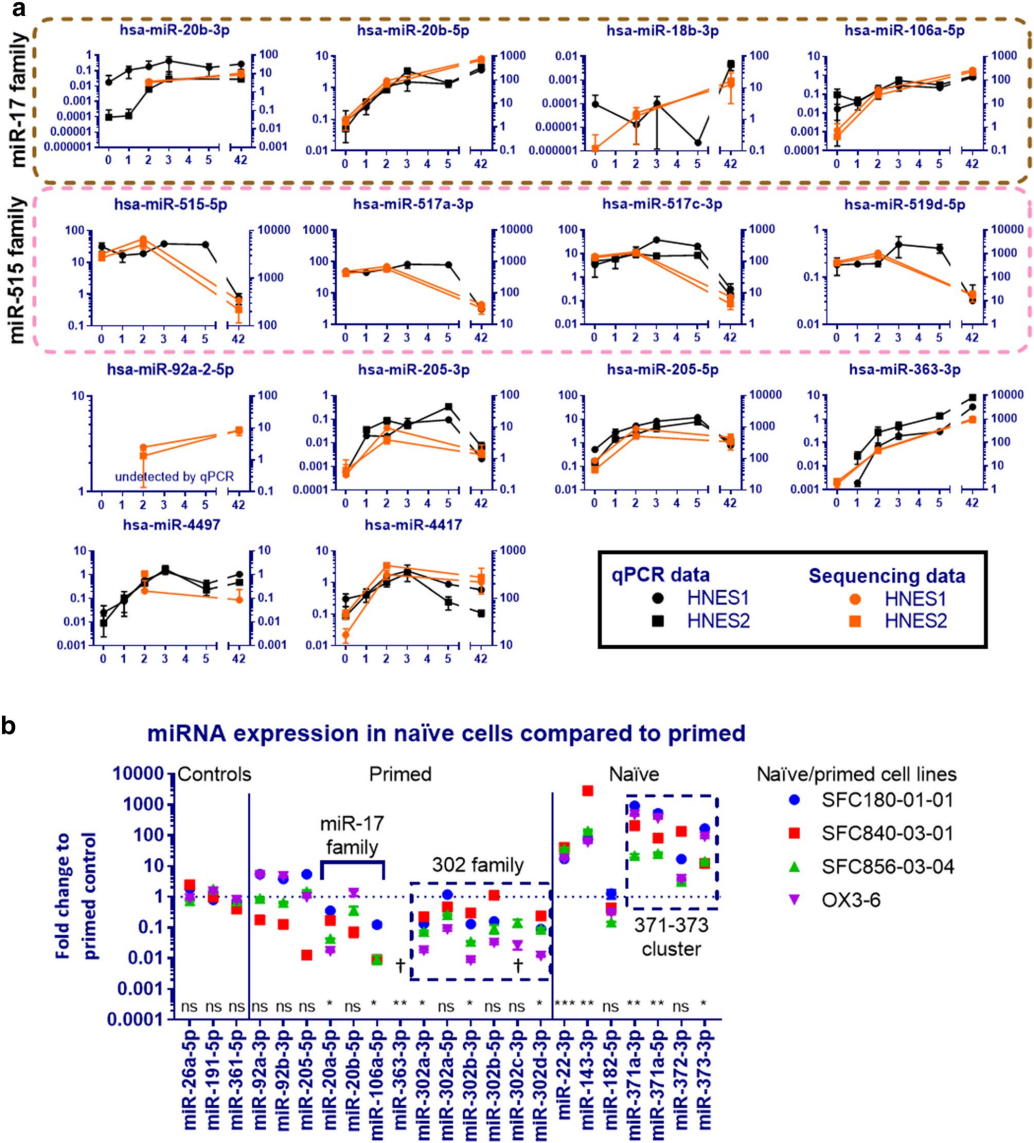
Validation of sequencing data. (**a**) Sequencing data is compared with qPCR data from the entire time course. The left Y axis represents 2^−ΔCt^ values obtained by qPCR, the right Y axis sequencing results expressed as reads per million (RPM) displayed in orange. Sequencing was performed on samples from day 0, 2 and 42 and the mean of 3 technical replicates ± standard deviation is plotted. The qPCR data is plotted as the mean of 2 or 4 replicates ± standard deviation and is normalised to the mean of three endogenous control miRNAs (miR-26a-5p, miR-191-5p, miR-361-5p). Expression of miR-515-3p, -517a-3p, -519d-5p were assessed by qPCR on HNES1 cells only. Other missing qPCR datapoints represent undetectable expression. MiRNAs in dashed boxes are members of the same family. (**b**) Differentially expressed miRNAs were investigated in reset naïve compared to conventional primed iPSC. Four iPSC lines were converted to the naïve state using 4iLA conditions and their miRNA expression was compared to their parental primed controls using RT-qPCR. Expression was normalised first to the mean of three endogenous control miRNAs (miR-26a-5p, miR-191-5p, miR-361-5p), then to their primed control (expressed as fold change or 2^−ΔΔCt^). The dagger (†) indicates where datapoints are missing due to cell lines which have high miRNA expression in the primed state but could not be plotted due to no expression in the naïve state. The miRNA miR-512-3p did not amplify exponentially by RT-qPCR and was excluded. Each symbol is the mean of 3 technical replicates ± standard deviation. A two-tailed ratio paired t-test of the mean 2^−ΔCt^ values of each cell line in naïve vs primed states informed significance. The test was also performed for undetected miRNAs (†), by calculating the 2^−ΔCt^ using a Ct value of 40 (the maximum number of cycles used in the qPCR reaction).

To elucidate the miRNA expression dynamics in more detail and validate our findings, we measured expression of day 2 upregulated miRNAs across the entire time course by RT-qPCR (Fig. 4a, Supplementary Fig. S3). MiR-363-3p as well as miR-17 family miRNAs miR-20b-3p, 20b-5p and -106a-5p are upregulated across the entire time course, whereas miR-205-3p, -205-5p, -4,417 and -4,497 peak during the first 5 days of transition. The miR-515 family members miR-515-5p, -517a-3p, -517c-3p and -519d-5p maintain high levels of expression over the first 5 days of the transition but are downregulated once the cells are fully primed. We observe an immediate upregulation of miR-17 family miRNAs during exit of the naïve state. Rapid upregulation has also been observed in mouse embryogenesis, where an intermediate pluripotent state (poised to become primed) with high miR-17 family expression has been identified^33^. In total, 44 miRNAs were assessed by qPCR.

The miRNAseq was performed using embryo-derived naïve cells. To validate naive and primed marker miRNAs, we used naive cells obtained by resetting from conventional hPSC. Human iPSC from 5 genetic backgrounds were converted to the naïve state according to the adapted Theunissen protocol (4iLA; omitting the GSK3 inhibitor) as previously published^10,25^. Three of the five converted lines were karyotypically normal and were used in all further experiments (Supplementary Fig. S2A). Naïve SFC856-03-04 line had minor abnormalities and was included in experiments as a fourth replicate but is to be interpreted with caution. Cells were confirmed as naïve by assessing gene expression (Fig. S2B).

The expression of the putative miRNA marker genes was assessed by qPCR (Fig. 4b). No significant difference in expression was observed for miR-92a-3p or -92b-3p, suggesting that these are not universal primed markers. No significant difference was also observed for miR-205-5p, which is in line with our observation of transient upregulation during the transition phase (Fig. 3b).

Two out of the three investigated miR-17 family miRNAs (miR-20a-5p and -106a-5p) as well as miR-363-3p and several miR-302 family miRNAs were significantly differentially expressed, confirming that these miRNAs are indeed markers of the primed state. The miRNAs miR-22-3p and -143-3p as well as the miR-371-373 cluster which were upregulated in naïve HNES cells were also upregulated in naïve 4iLA iPSC and can act as markers of the naïve state. However, the silencing of the miR-371–373 cluster during the naïve-primed transition is variable. MiR-182-5p was not significantly differentially expressed in 4iLA iPSC.

These results show, using two different naïve hPSC protocols, a diverse set of cell lines and two different methods, a set of state-associated human PSC miRNA markers that are more similar to mouse PSC than previously appreciated. Our novel miRNAseq dataset can be used for further comparisons to different naïve and primed hPSC or across species. The markers of naïve and primed states found here can be used for quality assessment when generating naïve cells. By using two very different strategies for generating naïve hPSC (directly derived HNES cells and reset 4iLA cells), we have removed many technical artefacts. It is still important to note, that both cell culture media use overlapping inhibitor conditions and that the observed naïve and primed miRNAs are in part generated by specific pathway inhibition rather than necessarily corresponding to a specific pluripotency state.

### Supplementary information

Supplementary information

## Data Availability

The miRNAseq data is available under the accession number GEO: GSE128260.
