# Supplementary material for: Profiling of naïve and primed human pluripotent stem cells reveals state-associated miRNAs
Source: Sci Rep. 2020 Jun 29;10:10542. doi: 10.1038/s41598-020-67376-w (PMC7324611; doi:10.1038/s41598-020-67376-w)
Supplement: Supplementary file 1 — Supplementary information [file 41598_2020_67376_MOESM1_ESM.pdf]

Title: Profiling of naïve and primed human pluripotent stem cells reveals state-associated miRNAs

Benjamin T. Dodsworth, Klas Hatje, Maria Rostovskaya, Rowan Flynn, Claas A. Meyer, Sally A. Cowley

Supplemental Materials and Methods

Reagents were from ThermoFisher unless stated otherwise.

Cell culture

The origins of the iPSC lines used in this study are described in Table S2, and the naïve hESC lines HNES1 and HNES2 cells were previously derived (Guo et al., 2016). Cells were grown in a humidified incubator at 37°C, 7% CO_2, 5% O_2. Primed hPSC were grown on geltrex precoated plates (A1413302) in E8 medium (A15169-01). Cells were fed daily by aspirating all medium and replacing it with fresh, room temperature medium. Routine expansions were performed at approximately 80% confluency, maintaining cell aggregates as previously described (Beers et al., 2012). Cells were thawed from large, karyotyped batches and kept in culture only for the duration of an experiment.

Naïve cells were grown on irradiated (30 gy) CF1 mouse embryonic fibroblasts (MEFs) (Millipore; PMEF-CFL) with daily media changes. Naïve iPSC were generated as previously described (Theunissen et al., 2014) with minor adaptations from subsequent publications: IM-12 from the original Theunissen *et al.* was omitted in 4iLA (Theunissen et al., 2016); 70% media changes were implemented (Collier et al., 2017). t2iLGoY for HNES cells was prepared as previously described (Guo et al., 2016). Naïve cells were split 1:1-1:3 every 3-4 days cells by PBS washing, adding 1 mL of Accumax (A7089; Sigma), incubating at 37°C for 4 minutes and dissociating by pipetting with a P1000. Naïve cells were more adherent to each other, yet more resilient when mechanically manipulated than conventional primed cells, so were vigorously dissociated. Cells were then diluted in 10% ES FBS (16141-079) in KO DMEM (10829-018) and spun at 400 x *g* for 5 minutes, resuspended and plated down on irradiated MEFs. To prime naïve cells, 1 – 5 x 10^4 cells/cm^2 were plated onto a well of a six well plate (precoated with geltrex for >1 h). Medium was switched to E8 after 48 hours.

To freeze, naïve cells were dissociated to single cells as discussed above. After centrifugation, cells were resuspended in their regular medium at 2 x 10^6 cells/mL for primed cells and 0.75 mL per dissociated well (6 well plate) for naïve cells. Then an equal volume of ice-cold 2X freeze medium was added. 2X freeze medium consisted of 20% DMSO (D2650; Sigma), 20% KO-DMEM (10829-018), 60% ES FBS (16141-079). Cells were briefly and gently mixed and aliquoted into 1 mL in pre-cooled (4°C) cryovials, transferred into pre-cooled (4°C) cryo-bombs (containing isopropanol; C1562-1EA; Sigma), stored overnight at -80°C, then transferred to -196°C nitrogen vapour storage.

To thaw cells, a vial was swirled in a 37°C stirred water bath and transferred to at least 4 volumes of PBS, spun at 400 x *g* for 5 minutes and resuspended in the appropriate medium containing 10 µM ROCKi. Primed cells were then seeded onto wells pre-treated with geltrex. Naïve cells were plated on 1 well of MEFs prepared as described above.

Karyotyping

DNA was extracted from pellets containing approximately 1 x 10^6 cells using the DNeasy blood and tissue kit (69506; Qiagen) following the manufacturer's instructions. DNA samples (500 ng in 10 µL) were processed by the Wellcome Trust Centre for Human Genetics, Oxford, using Illumina Human OmniExpress24 array (700,000 SNPs). Resulting image data files were analysed by GenomeStudio and Karyostudio software (Illumina) to assess genome integrity.

RT-qPCR and miRNAseq

RNA extractions from cell pellets (equal or less than 1 x 10^6 cells) were performed using the RNeasy mini kit (74104; Qiagen) or the miRNeasy mini kit (for the analysis of miRNAs; 217004; Qiagen), following the manufacturer's protocol.

Conventional RT-qPCR was performed using the High Capacity RNA to cDNA kit (4387406), TaqMan™ primer-probes (4331182) and TaqMan™ Gene Expression Master Mix (4369016) according to the manufacturer's protocol. Samples were run either in 96 well format (15 µL per well, in duplicate or triplicate) on a StepOnePlus (4376600) or in 384 well format (10 µL per well, quadruplets) on a Lightcycler 480 (05015243001; Roche Life Science). Plates were sealed with MicroAmp™ Optical Adhesive Film (4311971) and centrifuged at 1000 x *g* for 2 minutes before running. RNA from samples from the multilineage differentiation was extracted from cells with Reliaprep RNA Miniprep and up to 1µg was used for reverse transcription using Goscript Reverse Transcription system (both from Promega). Quantitative PCR was performed with GoTaq qPCR Master Mix (Promega) using Universal Probe Library (Roche) for detection. Primer sequences are listed below.

For miRNA RT-qPCR, the TaqMan advanced miRNA assay cDNA synthesis kit (A28007) was used in conjunction with TaqMan Advanced miRNA Assays (A25576) and TaqMan Fast Advanced Master Mix (4444557) according to the manufacturer's instructions. Reactions were performed in quadruplets in 384 well format (10 µL per well) in a 7900HT Fast Real-Time PCR System with 384-Well Block Module (4329001) or in triplicates in 96 well format (15 µL per well) on a StepOnePlus (4376600). Analysis was performed by normalising against the average Ct of the endogenous controls hsa-miR-191-5p, hsa-miR-361-5p and hsa-miR-26a-5p. Data is expressed as $2^{-\Delta\Delta ct}$ (Livak and Schmittgen, 2001).

Trilineage differentiation

To test differentiation potential, naïve hPSC were plated to Geltrex, then after 48 hours the medium was changed to E8 (produced in house). The cells were continuously cultured then in E8, being passaged when reached confluency.

Neuroectoderm differentiation was induced in N2B27 medium supplemented with 500nM LDN193189 (Cat. 1509, Axon) and 1uM A8301 (Tocris, Biotechne) for 10 days (Chambers et al., 2009, with modifications).

Definitive endoderm differentiation was performed according to Loh et al. 2014 (Loh et al., 2014). The cells were cultured in CDM2 medium (consisting of 1:1 mixture IMDM and F12, 2 mM L-Glutamine, 1% Chemically defined lipid concentrate (all from Gibco Thermo Fisher Scientific), 0.1% BSA or polyvinyl alcohol, 15µg/ml transferrin, 450µM monothioglycerol, 0.7µg/ml insulin (all from Sigma-Aldrich)) supplemented with 100ng/ml Activin A (produced in house), 100nM PI-103 (#2930, Tocris, Biotechne), 3µM CHIR99021, 10ng/ml FGF2 (produced in house), 3ng/ml BMP4 (Peprotech) for one day. The following supplements were applied for the next two days: 100ng/ml Activin A, 100nM PI-103, 20ng/ml FGF2, 250nM LDN193189.

Lateral mesoderm differentiation was done as in Loh et al. 2016 (Loh et al., 2016), the cells were cultured in CDM2 supplemented with 30ng/ml Activin A, 40ng/ml BMP4 (Milteniy Biotech), 6uM CHIR99021, 20ng/ml FGF2, 100nM PI-103 for 1 day, then the medium was supplemented with 1uM A8301, 30ng/ml BMP4 and 10uM XAV939 (Sigma-Aldrich) for two more days.

Flow cytometry

For surface marker staining, the cells were dissociated using Accutase and washed using PBS with 2% FCS. Incubation with directly conjugated antibodies (listed below) diluted in PBS with 2% FCS was done for 1 hour at +4°C, followed by washing and resuspending in PBS. For staining for intracellular markers, the cells were fixed with Fixation Buffer (00-8222-49, eBiosciences) for 30 min at +4°C, washed with Permeabilization Buffer (00-8333-56, eBiosciences) and further incubated with antibodies diluted with 5% donkey serum (Sigma-Aldrich) in Permeabilization buffer, for 1 hour at +4°C. The detection of staining was done using BD Fortessa LSR. The data analysis has been performed using FlowJo software.

Antibody	Cat. Number	Company	Dilution
PE conjugated mouse IgG2a anti-human CD184 (CXCR4)	555974	BD Pharmingen	1:25
APC conjugated goat polyclonal anti-human SOX17	IC1924A	RnD (Biotechne)	1:100
PE conjugated mouse IgG1 anti-human KDR	FAB357P-100	RnD (Biotechne)	1:25
BV421 conjugated mouse IgG2a anti-human PDGFRa	562799	BD Biosciences	1:50
Alexa647 conjugated mouse IgG1 anti-human SOX1	562224	BD Biosciences	1:200

mRNA	Forward (5’-3’)	Reverse (5’-3’)	Product size	UPL probe
SOX17	acgccgagttgagcaaga	tctgcctcctccacgaag	82	61
HHEX	cggacggtgaacgactaca	agaaggggctccagagtagag	76	61
LHX1	atgcaacctgaccgagaagt	caggtcgctaggggagatg	121	80
CER1	gccatgaagtacattgggaga	cacagccttcgtgggttatag	69	41
SOX1	accaggccatggatgaag	cttaattgctggggaattgg	67	37
PAX6	ggcacacacacattaacacactt	ggtgtgtgagagcaattctcag	71	9
BRN2	aataaggcaaaaggaaagcaact	caaaacacatcattacacctgct	72	57
FOXG1	atgatccccaagtcctcgtt	gtggtggttgtcgttctgg	69	64
HAND2	gaagaccgacgtgaaagagg	ttgctgctcactgtgctttt	72	17
FOXF1	cagcctctccacgcactc	cctttcggtcacacatgct	122	5
IRX3	ctctccctgctgggctct	ccaaggcactacagcgatct	130	70
MESP1	ctgttggagacctggatgc	cgtcagttgtcccttgtcac	76	27

Supplemental References

Beers, J., Gulbranson, D.R., George, N., Siniscalchi, L.I., Jones, J., Thomson, J.A., and Chen, G. (2012). Passaging and colony expansion of human pluripotent stem cells by enzyme-free dissociation in chemically defined culture conditions. *Nat Protoc* **7**, 2029-2040.

Livak, K.J., and Schmittgen, T.D. (2001). Analysis of relative gene expression data using real-time quantitative PCR and the 2(-Delta Delta C(T)) Method. *Methods* **25**, 402-408.

Subramanian, A., Tamayo, P., Mootha, V.K., Mukherjee, S., Ebert, B.L., Gillette, M.A., Paulovich, A., Pomeroy, S.L., Golub, T.R., Lander, E.S., et al. (2005). Gene set enrichment analysis: a knowledge-based approach for interpreting genome-wide expression profiles. *Proc Natl Acad Sci U S A* **102**, 15545-15550.

Xu, J., Li, C.X., Li, Y.S., Lv, J.Y., Ma, Y., Shao, T.T., Xu, L.D., Wang, Y.Y., Du, L., Zhang, Y.P., et al. (2011). MiRNA-miRNA synergistic network: construction via co-regulating functional modules and disease miRNA topological features. *Nucleic Acids Res* **39**, 825-836.

Chambers, S. M., Fasano, C. A., Papapetrou, E. P., Tomishima, M., Sadelain, M. and Studer, L. (2009). Highly efficient neural conversion of human ES and iPS cells by dual inhibition of SMAD signaling. *Nature biotechnology* **27**, 275-280.

Loh, K. M., Ang, L. T., Zhang, J., Kumar, V., Ang, J., Auyeong, J. Q., Lee, K. L., Choo, S. H., Lim, C. Y., Nichane, M., et al. (2014). Efficient endoderm induction from human pluripotent stem cells by logically directing signals controlling lineage bifurcations. *Cell Stem Cell* **14**, 237-252.

Loh, K. M., Chen, A., Koh, P. W., Deng, T. Z., Sinha, R., Tsai, J. M., Barkal, A. A., Shen, K. Y., Jain, R., Morganti, R. M., et al. (2016). Mapping the Pairwise Choices Leading from Pluripotency to Human Bone, Heart, and Other Mesoderm Cell Types. *Cell* **166**, 451-467.

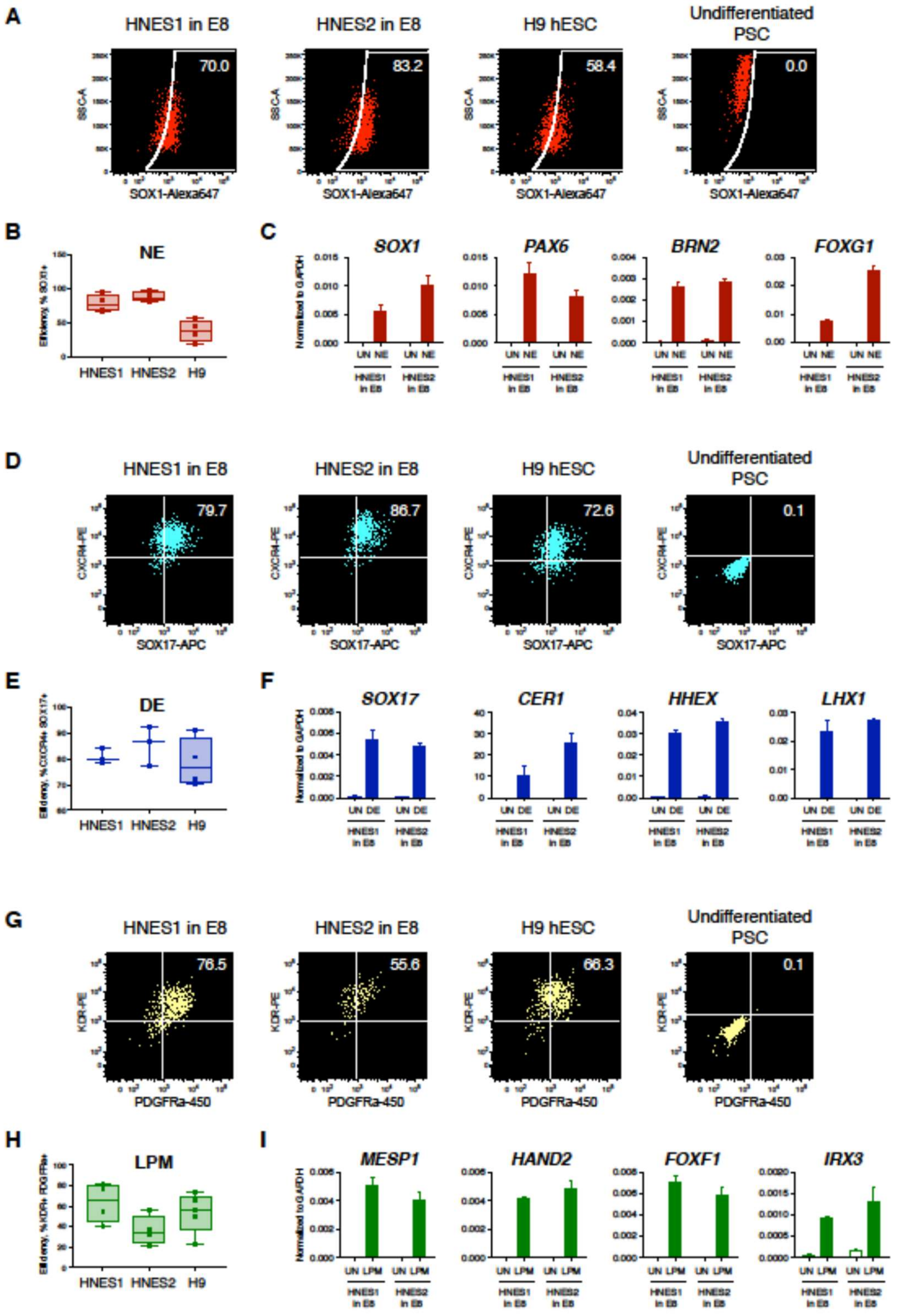

Figure S1. Multilineage differentiation of human naïve PSC after culturing in E8. Multilineage differentiation was performed with HNES1 and HNES2 after adaptation to E8 conditions, and conventional H9 hES. Neuroectoderm induction was assessed (A, B) using flow cytometry for SOX1, shown as dot plots after one representative experiment (A) and a summary of several independent experiments (B); and (C) by qRT-PCR for markers. Definitive endoderm differentiation was confirmed (D, E) using flow cytometry for CXCR4 and SOX17, shown as dot plots after one representative experiment (D) and a summary of several independent experiments (E); and (F) by qRT-PCR for markers. Induction of lateral plate mesoderm was quantified (G, H) using flow cytometry for KDR and PDGFRa, shown as dot plots after one representative experiment (G) and a summary of several independent experiments (H); and (I) by qRT-PCR for markers. NE – neuroectoderm, DE – definitive endoderm, LPM – lateral plate mesoderm, SSC – side scatter.

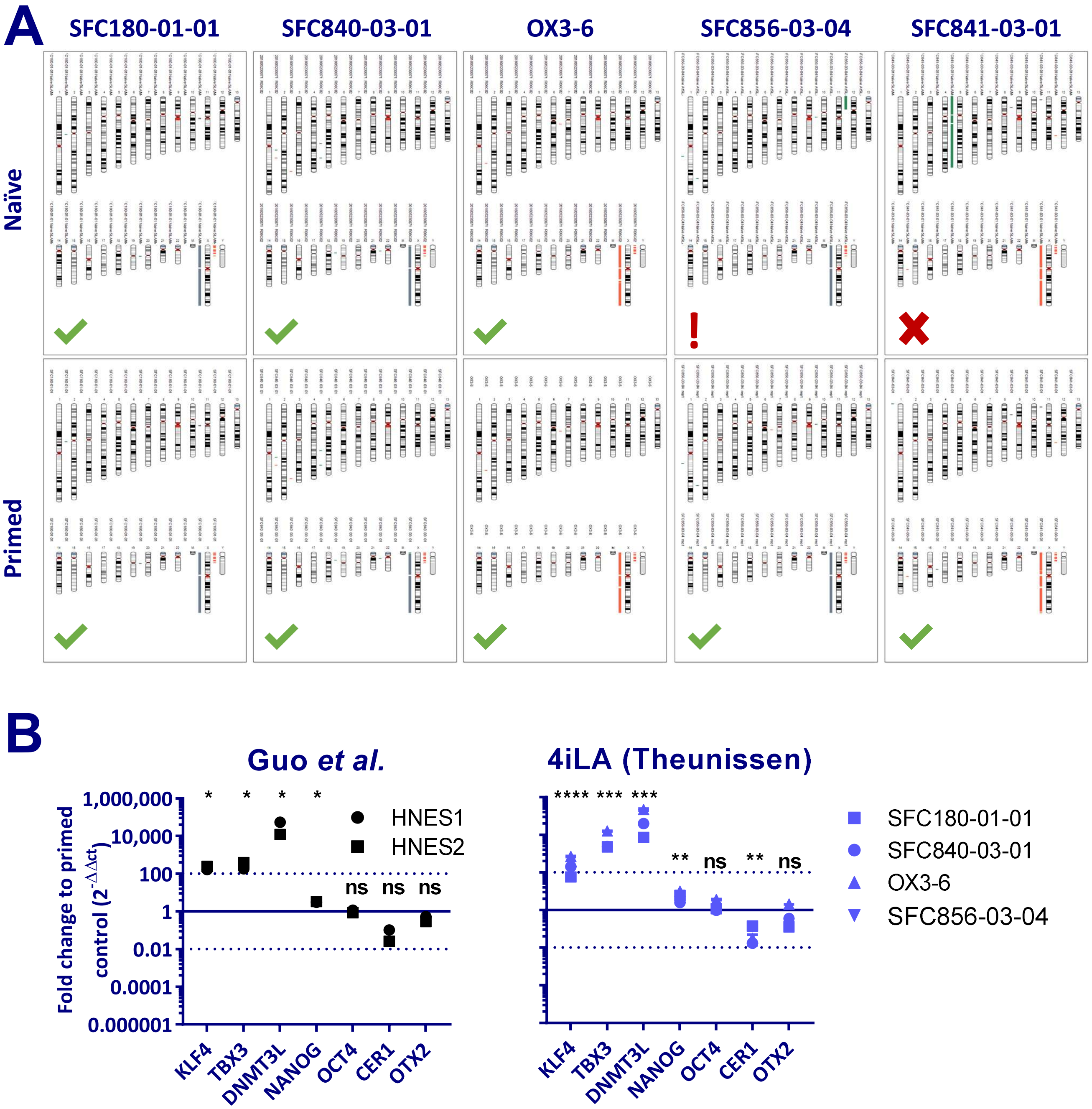

Figure S2: Quality control of cell lines. (A) High levels of karyotypic abnormalities have been reported with naïve hPSC (Guo et al., 2016; Kilens et al., 2018; Pastor et al., 2016; Theunissen et al., 2014), so large stocks were frozen and tested for karyotypic abnormalities by SNP array. Ticks indicate a karyotypically normal profile. The naïve SFC856-03-04 line had a duplication in the small arm of chromosome 12 and was included in experiments as a fourth replicate but is to be interpreted with caution. Naïve SFC841-03-01 had a duplication of the entire chromosome 5 and was therefore not included in further experiments. (B) To assess naivety, gene expression of naïve hPSC were compared their primed control by $2^{-\Delta\Delta Ct}$. A panel of naïve (*KLF4, TBX3, DNMT3L*), shared pluripotency (*NANOG, OCT4*) and primed genes (*CER1, OTX2*) were chosen. Datapoints represent the mean expression of each cell line with error bars showing the standard deviation of 3 technical replicates. Data normalised first to housekeeping genes (*B2M*, *RPL13A*), then to the primed control using the $2^{-\Delta\Delta ct}$ method. A two-tailed ratio paired t-test of the $2^{-\Delta ct}$ values of biological replicates (mean of each cell line) informed significance.

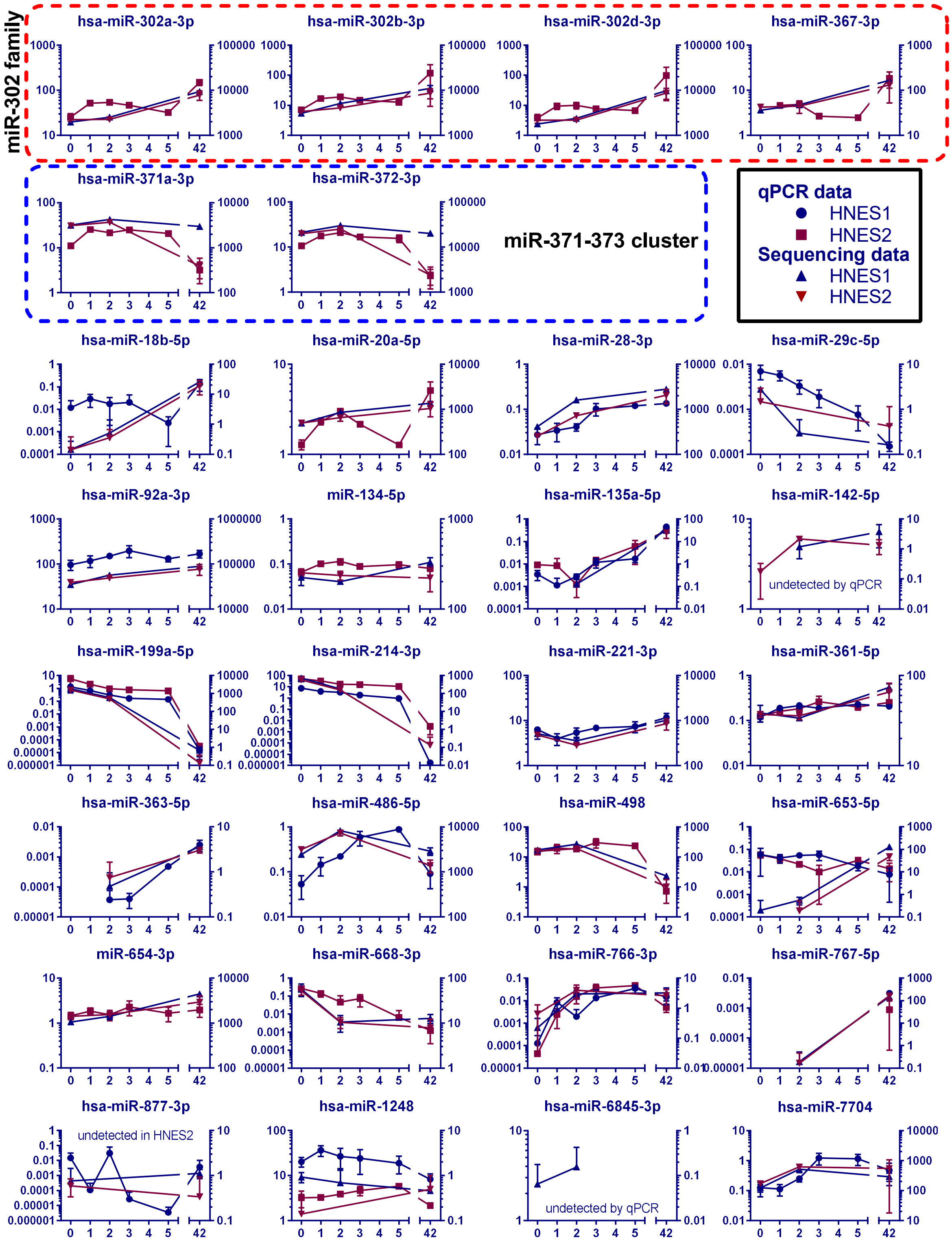

Figure S3: Sequencing data compared with qPCR results. The left Y axis represents $2^{-\Delta Ct}$ values obtained by qPCR, the right Y axis sequencing results expressed as reads per million (RPM) displayed as triangles. Sequencing was performed on samples from day 0, 2 and 42 and the mean of 3 technical replicates ± standard deviation is plotted. The qPCR data is plotted as the mean of 2 or 4 replicates ± standard deviation. MiRNAs in dashed boxes are in the same family and miRNAs in solid lined boxes are in the same cluster. The miRNAs hsa-miR-6845-3p, hsa-miR-142-5p, hsa-miR-6845-3p, hsa-miR-92a-2-5p were not detected by qPCR but were picked up by sequencing. Expression of miR-302 family, -371-373 cluster, -18b-5p, -20a-5p, -28-3p, -29c-5p, -92a-3p, -134-5p, -214-3p, -221-3p, -363-5p, -486-5p, -498, -654-3p, -668-3p, -767-5p, -6854-3p and -7704 were assessed by qPCR in one cell line only. Other missing qPCR datapoints represent undetectable expression. MiRNAs in dashed boxes are members of the same family.

NAME	SIZE	NES	NOM p-val	FDR	Pass
TBX3_MIRNA BINDING_INHIBITION_UPSTREAM	14	1.771	1.47E-03	**0.242**	Yes
AKAP13_MIRNA BINDING_UNSPECIFIED_UPSTREAM	17	1.724	3.36E-03	0.334	No
ABCG4_MIRNA BINDING_INHIBITION_UPSTREAM	10	1.732	4.46E-04	0.345	No
EGLN3_MIRNA BINDING_UNSPECIFIED_UPSTREAM	12	1.634	1.68E-02	0.434	No
VAPA_MIRNA BINDING_INHIBITION_UPSTREAM	9	1.635	1.87E-02	0.436	No
MAP3K8_MIRNA BINDING_UNSPECIFIED_UPSTREAM	8	1.637	1.84E-02	0.439	No
FGD1_MIRNA BINDING_UNSPECIFIED_UPSTREAM	7	1.635	1.14E-02	0.443	No
HSPA1B_MIRNA BINDING_UNSPECIFIED_UPSTREAM	22	1.638	1.11E-02	0.444	No
ATP1A2_MIRNA BINDING_UNSPECIFIED_UPSTREAM	9	1.655	8.99E-03	0.450	No
TNRC6A_MIRNA BINDING_UNSPECIFIED_UPSTREAM	6	1.628	2.15E-03	0.451	No

Table S1: Gene set enrichment analysis. Many miRNAs cause only a minor difference individually but synergistically with other miRNAs can downregulate genes effectively (Xu et al., 2011). To uncover which genes are most heavily regulated by miRNAs during the exit from naïve pluripotency, a gene set enrichment analysis (GSEA) was performed. Briefly, each gene was assigned all miRNAs which bind to its mRNA *in silico*. The gene set enrichment analysis then lists genes which have an enrichment of binding sites for miRNAs upregulated at day 2 of the differentiation compared to naïve cells. Due to the large amount of *in silico* binding partners of miRNAs and high frequency of false positives, the analysis is statistically not very powerful and the threshold for discovery is commonly set at a false discovery rate of 0.25 and below (Subramanian et al., 2005). In this analysis, one gene was below the discovery threshold, *TBX3*. *TBX3* was heavily downregulated during the first days of differentiation (Fig. 2), suggesting that miRNAs might be involved in dismantling the naïve pluripotency network.

The amount of miRNAs which bind the target gene *in silico* are listed in “size”. The nominal p-value (NOM p-val) estimates significance only for a single gene set. Unlike the p-value, the false discovery rate (FDR) is adjusted for gene set size and multiple hypothesis testing and is therefore a more relevant measure. The normalised enrichment score (NES) accounts for differences in gene set size and in correlations between gene sets and the expression dataset, and is a measure of enrichment (Subramanian et al. 2005).

line name	alternative name	reprogramming method	PMID
OX3-6		Sendai (Nakanishi)	29604226
SFC180-01-01		Sendai (CytoTune 2)	28827786
SFC840-03-01	AH017-11	Sendai (CytoTune 1)	26905200
SFC841-03-01	OX1-61	Sendai (CytoTune 1)	27097283
SFC856-03-04		Sendai (CytoTune 2)	28827786

Table S2: Origins of iPSC lines used in this publication.
